# The Metropolitan Hospital

**Published:** 1914-07-25

**Authors:** J. Courtney Buchanan

**Affiliations:** Secretary and House Governor. Metropolitan Hospital, Kingsland Road, N.E.


### THE EDITOR'S LETTER-BOX.

### The Metropolitan Hospital.

*To the Editor of* The Hospital.

Sir,—I beg to acknowledge with best thanks your kindness in giving publicity to the needs of the Metropolitan Hospital in your issue of the 11th instant.

There is no doubt that we have been through a period of great financial difficulty, and we have only recently begun to get some slight relief.

This must be due in large measure to the publicity given to our needs by the Press, and for this generosity the committee are sincerely grateful. I have the honour to be, Sir, your obedient servant,

J. Courtney Buchanan,
Secretary and House Governor.

Metropolitan Hospital, Kingsland Road, N.E.,
July 16, 1914.

